# Assessing environmental enteric dysfunction via multiplex assay and its relation to growth and development among HIV-exposed uninfected Tanzanian infants

**DOI:** 10.1371/journal.pntd.0011181

**Published:** 2023-03-21

**Authors:** Jacqueline M. Lauer, Miles A. Kirby, Alfa Muhihi, Nzovu Ulenga, Said Aboud, Enju Liu, Robert K. M. Choy, Michael B. Arndt, Jianqun Kou, Wafaie Fawzi, Andrew Gewirtz, Christopher R. Sudfeld, Karim P. Manji, Christopher P. Duggan

**Affiliations:** 1 Department of Health Sciences, College of Health & Rehabilitation Sciences: Sargent College, Boston University, Boston, Massachusetts, United States of America; 2 Department of Global Health and Population, Harvard T.H. Chan School of Public Health, Boston, Massachusetts, United States of America; 3 Management and Development for Health, Dar es Salaam, Tanzania; 4 Department of Microbiology and Immunology, Muhimbili University of Health and Allied Sciences, Dar es Salaam, Tanzania; 5 Division of Gastroenterology, Hepatology and Nutrition, Boston Children’s Hospital, Boston, Massachusetts, United States of America; 6 Institutional Centers for Clinical and Translational Research, Boston Children’s Hospital, Boston, Massachusetts, United States of America; 7 PATH, Center for Vaccine Innovation and Access, Seattle, Washington, United States of America; 8 Institute for Health Metrics and Evaluation, University of Washington, Seattle, Washington, United States of America; 9 Institute for Biomedical Sciences, Georgia State University, Atlanta, Georgia, United States of America; 10 Department of Nutrition, Harvard T.H. Chan School of Public Health, Boston, Massachusetts, United States of America; 11 Department of Pediatrics and Child Health, Muhimbili University of Health and Allied Sciences, Dar es Salaam, Tanzania; University of Washington, UNITED STATES

## Abstract

**Background:**

Environmental enteric dysfunction (EED) may contribute to poor growth and development in young children. While validated EED biomarkers are currently lacking, multiplex assays are able to capture multiple domains of the condition. The purpose of this exploratory study was to examine the relationship between biomarkers of EED and subsequent growth and development among Tanzanian HIV-exposed uninfected (HEU) infants.

**Methodology:**

We enrolled 467 infants of mothers living with HIV who had participated in a trial of vitamin D_3_ supplementation during pregnancy. Infant serum samples collected at 6 weeks (n = 365) and 6 months (n = 266) were analyzed for anti-flagellin and anti-lipopolysaccharide (LPS) IgA and IgG via ELISA as well as the 11-plex Micronutrient and EED Assessment Tool (MEEDAT), which incorporates two biomarkers of EED [intestinal fatty acid-binding protein (I-FABP) and soluble CD14 (sCD14)]. Outcomes were 12-month growth [length-for-age z-score (LAZ), weight-for-length z-score (WLZ), and weight-for-age z-score (WAZ)] and development [Caregiver Reported Early Development Instruments (CREDI) z-scores] and were assessed using linear regression.

**Findings:**

In primary analyses, higher quartiles of 6-month anti-LPS IgG concentrations were significantly associated with lower LAZ at 12 months (p_trend_ = 0.040). In secondary analyses, higher log_2_-transformed 6-week anti-flagellin IgA and 6-month anti-LPS IgA concentrations were significantly associated with lower LAZ at 12 months. No associations were observed between I-FABP or sCD14 and infant growth. However, higher log_2_-transformed 6-week sCD14 concentrations were significantly associated with lower overall CREDI z-scores, while higher log_2_-transformed 6-month I-FABP concentrations were significantly associated with higher overall CREDI z-scores.

**Conclusions:**

Unlike anti-flagellin and anti-LPS Igs, MEEDAT’s biomarkers of EED (I-FABP and sCD14) were not associated with subsequent linear growth among HEU infants in Tanzania. The relationship between EED and infant development warrants further study.

## Author summary

Growth failure can begin in utero and worsen during early infancy; environmental enteric dysfunction (EED) has been hypothesized as an important causal factor. EED is an acquired, subclinical condition of the small intestine associated with chronic exposure to an unhygienic environment where enteric pathogens persist. Histologic features include mucosal inflammation, villous blunting, altered barrier integrity, and reduced intestinal absorptive capacity. Despite EED’s purported link to poor health and nutrition outcomes in young children in low- and middle- income countries (LMICs), our knowledge of EED is relatively limited and is hindered by the lack of validated biomarkers across EED’s various domains. In this exploratory study, we examined the relationship between biomarkers of EED, including ones in the 11-plex Micronutrient and EED Assessment Tool (MEEDAT), and growth and development in HIV-exposed uninfected (HEU) Tanzanian infants. Overall, we found that certain anti-flagellin and anti-LPS Igs assessed at 6 weeks and 6 months were significantly associated with reduced linear growth at 12 months. EED biomarkers in MEEDAT were significantly associated with subsequent developmental, but not growth, outcomes.

## Introduction

Stunting, or a length/height-for-age z-score (LAZ/HAZ) < –2 using the World Health Organization (WHO) Child Growth Standards median, affects an estimated 149 million children [1]. In particular, HIV-exposed uninfected (HEU) infants are at an increased risk of stunting compared with their HIV-unexposed uninfected (HUU) peers, likely due to both maternal sociodemographic differences and greater risk of adverse birth outcomes associated with infant growth [2, 3]. To date, stunting has been linked to numerous short- and long-term consequences, including an increased risk of morbidity and mortality, diminished cognitive development, poorer educational outcomes, and lower economic productivity in adulthood [4].

Despite its large global burden, the etiology of stunting, including the role of chronic, asymptomatic gastrointestinal permeability and inflammation, i.e., environmental enteric dysfunction (EED), is not well understood [5]. Although EED is practically ubiquitous among populations living in low- and middle-income countries (LMICs) [6–8], its links to poor growth and development outcomes remain in question. Further, several underlying mechanisms, including lower nutrient intake due to anorexia, nutrient malabsorption due to reduced epithelial surface area, increased intestinal permeability with subsequent bacterial translocation and immune stimulation, and higher energy expenditure and growth-inhibiting effects due to chronic inflammation, have been proposed [9–12].

We previously observed that serum concentrations of the EED biomarkers anti-flagellin and anti-lipopolysaccharide (LPS) immunoglobulins (Igs) were negatively associated with growth [13, 14] but positively associated with cognitive development [15]. However, such serum biomarkers, while relatively simple to collect, are limited by their ability to assess only a single functional domain of the condition and are typically measured individually using conventional ELISA kits which, while accurate, are labor-intensive and expensive to run. Thus, multiplex assays, which are capable of measuring multiple analytes simultaneously in a single sample, hold promise. In particular, the 11-plex Micronutrient and EED Assessment Tool (MEEDAT), co-developed by PATH and Quansys Biosciences, incorporates two sera-based markers of EED [intestinal fatty acid-binding protein (I-FABP) and soluble CD14 (sCD14)] and two growth factors [insulin-like growth factor 1 (IGF-1) and fibroblast growth factor 21 (FGF21)]. These markers were added to the Q-Plex Human Micronutrient Array (7-plex) that measures micronutrient biomarkers [ferritin, soluble transferrin receptor (sTfR), retinol-binding protein 4 (RBP4), and thyroglobulin (Tg)], acute and chronic inflammatory biomarkers [C-reactive protein (CRP) and α1-acid glycoprotein (AGP)], and histidine-rich protein 2 (HRP2) [16].

Currently, the ability of MEEDAT to predict outcomes such as poor growth and development is not well established. The primary goal of this exploratory study was to examine the relationship between biomarkers of EED, including anti-flagellin and anti-LPS IgA and IgG as well as those in MEEDAT, and growth and developmental outcomes among HEU Tanzanian infants at risk of EED.

## Methods

### Ethics statement

The study was approved by Boston Children’s Hospital Institutional Review Board (reference no. IRB-P00033836), the Tanzanian National Health Research Ethics Sub-Committee (reference no. NIMR/HQ/R.8a/Vol.IX/3309), and the Muhimbili University of Health and Allied Science (MUHAS) Research and Ethics Committee (reference no. MUHAS-REC-11-2019-065). Written informed consent in Kiswahili was obtained from all participants in the parent trial.

### Description of cohort

Infants in this exploratory, prospective cohort study were participants in a randomized, double-blind, placebo-controlled trial conducted between 2015 and 2019 assessing the effect of vitamin D_3_ supplementation for pregnant and lactating women living with HIV on the risk of maternal HIV progression, fetal growth restriction, and infant stunting (ClinicalTrials.gov identifier: NCT02305927). Pregnant women were eligible for participation in the parent trial if they were ≥ 18 years, 12–27 weeks gestation, living with HIV and receiving ART, and had normal albumin-adjusted calcium concentrations (<2.6 mmol/L). Women were excluded if they intended to leave the study area within 2 years after study enrollment, were enrolled in another clinical trial, or refusal to provide informed consent. Women were followed-up at monthly clinic visits during pregnancy, at delivery, and then with their child at monthly postpartum clinic visits until infants reached 12 months of age. More detailed methods for the parent trial have previously been published [17]. Overall, the trial found no effect of maternal vitamin D_3_ supplementation on maternal HIV progression, maternal death, or subsequent infant stunting [18].

At baseline, researchers administered a questionnaire to collect data on sociodemographic characteristics. Birth outcomes were recorded by trained research staff. All infants received an HIV-1 DNA PCR test at 6 weeks and 12 months of age. In follow-up visits, infant length was assessed in triplicate using a rigid length board with an adjustable foot piece to 1-mm precision (SECA, Hamburg, Germany). Weight was measured in triplicate to the nearest 5 g using a digital scale (SECA, Hamburg, Germany). At 12 months, infant development was assessed using the Caregiver Reported Early Development Instruments (CREDI). CREDI is a population-level, caregiver-reported measure of early childhood development for children under 3 which was originally developed in Tanzania [19] and is considered to be a valid, acceptable, and reliable instrument for use in LMIC contexts based on a 17-country study [20, 21]. Blood samples were collected at 6 weeks, 6 months, and 12 months of age, and serum aliquots were stored at -80°C until shipped on dry ice for laboratory analyses.

A total of 2,300 pregnant women were randomized in the parent trial, and 720 with an available serum sample at 32 weeks gestation were randomly selected for participation in the EED sub-study using SAS (PROC SURVEYSELECT, version 9.4). This sample size was calculated to have >90% power to detect an effect size of 0.02 (or 2.0% of the variance in the outcome attributed to a given biomarker), assuming α = 0.05, 1 tested predictor, 10 additional independent covariates, and losses to follow-up of ~20% (G*Power software, Düsseldorf, Germany). Of these, 511 women had an infant with at least one serum sample at 6 weeks and/or 6 months.

### Laboratory analyses

Serum samples were analyzed for anti-flagellin and anti-LPS IgA and IgG at the Gewirtz Laboratory at Georgia State University using an ELISA as previously described [22]. Briefly, microtiter plates were coated with purified *Escherichia coli* flagellin (100 ng/well) or *Escherichia coli* LPS (1 ug/well). Serum samples were diluted at 1:500 and applied to wells coated with flagellin or LPS. After incubation and washing, the wells were incubated with anti-human IgA (KPL, Catalog No. 14-10-01) or IgG (GE Healthcare, Catalog No. 375112) coupled to a horseradish peroxidase. The quantification of total immunoglobulins was performed with the use of the colorimetric peroxidase substrate tetramethylbenzidine, and absorbance was read at 450nm and 540nm using an enzyme-linked immunosorbent assay plate reader. Concentrations were measured and reported as optical density (OD) units.

Serum samples were also analyzed using the Micronutrient and EED Assessment Tool (MEEDAT) at Quansys Biosciences (Logan, Utah) using previously reported methods [23]. Samples were diluted at 1:10 with the appropriate Quansys sample dilution buffer. Polypropylene low-binding 96-well plates were used to prepare the samples and standards prior to loading the Q-Plex plate. Each dilution was measured in duplicate for a total of two wells per sample. An image with a 270 second exposure time was captured using a Q-View Imager LS and Q-View Software. Concentrations of luminescent units or pixel intensity units were then measured by the Q-View Software.

### Statistical analyses

Descriptive characteristics were calculated and presented as mean ± SD or n (%). LAZ, WLZ, and WAZ, were calculated using the WHO Multicenter Growth Reference Study growth standards [24]. Median (IQR) values were calculated for each biomarker, and Pearson correlation coefficients were calculated to assess relationships between biomarker concentrations. Norm-referenced standardized z-scores for motor development, language development, cognitive development, and overall development, were calculated using the CREDI Scoring App version 0.1 (https://credi.shinyapps.io/Scoring_App/). All other statistical analyses were carried out using STATA 15 software (Stata Corp, College Station, TX).

For primary analyses, biomarker concentrations were categorized into quartiles, with the lowest quartile as the reference. The associations between biomarker concentrations at 6 weeks and 6 months and infant LAZ, WLZ, WAZ, and CREDI z-scores at 12 months were assessed using linear regression models with robust standard errors to address deviations from normality. Models included adjustments for potential confounders and factors associated with LAZ at 12 months, including household wealth quintile, maternal age (18–24, 25–35, 35+ years), continuous maternal height, maternal education (none, primary, secondary/advanced), maternal marital status (married/cohabitating, single, widowed/divorced separated), infant sex (male/female), continuous infant birth weight, continuous infant age at specimen collection, clinic site, and study arm (placebo or vitamin D_3_). A household wealth index was constructed using a principal component analysis (PCA) of household ownership of assets (electricity, couch, television, refrigerator, fan, bicycle, and car) and was divided into quintiles for analysis. P-values for trend were calculated regressing the median of each biomarker quartile. For secondary growth and CREDI analyses, biomarkers were kept as continuous exposures transformed to the log base 2 scale, interpreted as the effect of doubling the biomarker concentration. The absence of multi-collinearity among predictors was verified using variance inflation factor (VIF). For all analyses, a *p*-value < 0.05 was considered statistically significant.

## Results

**Fig 1:**
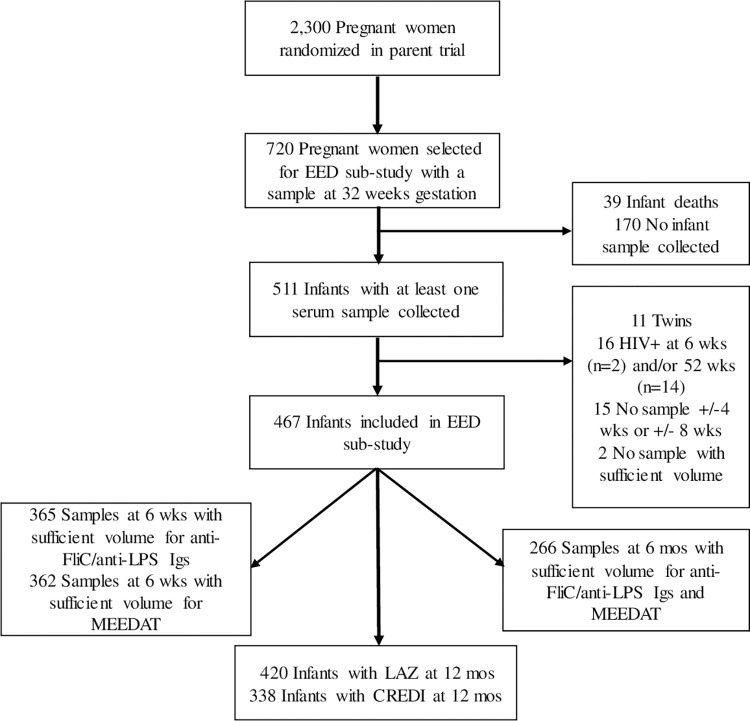
Flow diagram for study. Abbreviations: CREDI, Caregiver Reported Early Development Instruments; EED, environmental enteric dysfunction; FliC, flagellin; Ig, immunoglobulin; LAZ, length-for-age z-score; LPS, lipopolysaccharide; MEEDAT, Multi-Micronutrient and Environmental Enteric Dysfunction Assessment Tool.

The flow diagram for the study can be found in **Fig 1**. Infants were excluded if they were a twin (n = 11) or if the infant was documented to have HIV infection (n = 16). Fifteen infants were excluded due to their sample being outside the visit window of +/-4 weeks for the 6-week sample or +/- 8 weeks for the 6-month sample, and 2 did not have a sample with sufficient volume. In total, 467 infants were included in this study; 365 of whom had a serum sample at 6 weeks and 266 of whom had a serum sample at 6 months.

### Descriptive characteristics

**Table 1** presents descriptive characteristics for 467 HEU infants and their mothers from the baseline visit in pregnancy. On average, mothers were 31 years old and ~158 cm tall, and the majority (~77%) were married or cohabitating. Slightly over half (~53%) of infants were male. At 12 months, mean LAZ, WLZ, and WAZ were -1.72 ± 1.45, 0.60 ± 1.42, and -0.44 ± 1.18, respectively. **S1 Table** shows the comparison of descriptive characteristics among the 6-week study sample (n = 365), the 6-month study sample (n = 266), and the full EED sub-study sample (n = 720).

**Table 1:** Descriptive characteristics for 467 infants and their mothers in Dar es Salaam, Tanzania.

	Mean ± SD or n (%)
**Maternal characteristics**	
Age, years	
[Table-fn t001fn001][Table-fn t001fn001][Table-fn t001fn001][Table-fn t001fn001]18–24	67 (14.4)
[Table-fn t001fn001]25–35	291 (62.3)
[Table-fn t001fn001]35+	109 (23.3)
Height, cm	158.1 ± 6.2
Education	
[Table-fn t001fn001] No formal education	59 (12.6)
[Table-fn t001fn001]Primary	259 (55.5)
[Table-fn t001fn001]Secondary/advanced	148 (31.7)
[Table-fn t001fn001]Missing	1 (0.2)
Marital status	
[Table-fn t001fn001]Married/cohabitating	359 (76.9)
[Table-fn t001fn001]Single	94 (20.1)
[Table-fn t001fn001]Widowed/divorced/separated	14 (3.0)
Regimen	
[Table-fn t001fn001]Placebo	240 (51.4)
[Table-fn t001fn001]Vitamin D_3_	227 (48.6)
**Infant characteristics**	
Male	248 (53.1)
Low birth weight (<2500 g)	23 (4.9)
Stunted at 12 months (LAZ < -2)^1^	192 (45.7)
Wasted at 12 months (WLZ < -2)^2^	11 (2.9)
Underweight at 12 months (WAZ < -2)^3^	36 (9.5)

Abbreviations: LAZ, length-for-age *z*-score, WAZ, weight-for-age *z*-score, WLZ, weight-for-length *z*-score

^1^n = 420

^2^n = 376

^3^n = 380

### Correlations among biomarkers

**Table 2** shows Pearson correlation coefficients of log_2_-transformed biomarker concentrations at 6 weeks and 6 months, including anti-flagellin and anti-LPS Igs and MEEDAT biomarkers. Correlations among anti-flagellin and anti-LPS Igs were generally high, ranging from 0.736 between anti-flagellin IgG and anti-LPS IgG (p<0.001) to 0.098 between anti-flagellin IgG and anti-LPS IgA at 6 weeks and from 0.853 between anti-flagellin IgA and anti-LPS IgA (p<0.001) and 0.274 between anti-flagellin IgG and anti-LPS IgA (p<0.001) at 6 months.

**Table 2:** Correlation coefficient matrix of biomarker concentrations at 6 weeks and 6 months of age^1^.

	n	Median (IQR)	FliC IgA	FliC IgG	LPS IgA	LPS IgG	sCD14	I-FABP	AGP	CRP	IGF-1	FGF21
6 weeks												
FliC IgA (OD)	365	0.47 (0.38, 0.57)	1.000									
FliC IgG (OD)	365	1.00 (0.80, 1.25)	0.229***	1.000								
LPS IgA (OD)	365	0.31 (0.23, 0.40)	0.701***	0.098	1.000							
LPS IgG (OD)	365	0.84 (0.61, 1.11)	0.303***	0.736***	0.374***	1.000						
sCD14 (ng/mL)	362	1829.46 (1404.62, 2287.57)	0.104*	-0.052	0.173***	-0.062	1.000					
I-FABP (pg/mL)	362	1094.62 (732.21, 1634.80)	-0.005	-0.155**	0.046	-0.145**	0.367***	1.000				
AGP (g/L)	362	0.83 (0.61, 1.26)	0.125*	0.007	0.188***	-0.047	0.616***	0.182***	1.000			
CRP (mg/L)	362	0.36 (0.00, 4.40)	0.119*	-0.055	0.123*	-0.070	0.367***	0.079	0.715***	1.000		
IGF-1 (ng/mL)	362	66.48 (32.24, 117.92)	-0.065	-0.172*	-0.029	-0.168**	0.098	0.214***	-0.220***	-0.322***	1.000	
FGF21 (pg/mL)	362	88.90 (47.94, 174.50)	-0.053	-0.085	-0.053	-0.115*	0.187***	0.209***	0.148**	0.056	-0.026	1.000
6 months												
FliC IgA (OD)	266	0.70 (0.54, 0.90)	1.000									
FliC IgG (OD)	266	1.30 (0.96, 1.80)	0.293***	1.000								
LPS IgA (OD)	266	0.45 (0.34, 0.65)	0.853***	0.274***	1.000							
LPS IgG (OD)	266	1.19 (0.78, 1.71)	0.426***	0.787***	0.493***	1.000						
sCD14 (ng/mL)	266	2200.61 (1683.76, 2876.08)	0.044	-0.216***	0.037	-0.224***	1.000					
I-FABP (pg/mL)	266	566.21 (411.72, 856.65)	0.085	-0.176**	0.092	-0.169**	0.436***	1.000				
AGP (g/L)	266	1.05 (0.83, 1.43)	0.071	0.031	0.127*	0.014	0.410***	0.147*	1.000			
CRP (mg/L)	266	0.29 (0.08, 1.54)	0.109	0.062	0.122*	0.030	0.338***	0.039	0.688***	1.000		
IGF-1 (ng/mL)	266	20.74 (9.64, 44.52)	-0.039	-0.166**	-0.039	-0.200**	0.192**	0.246***	-0.127*	-0.119	1.000	
FGF21 (pg/mL)	266	270.00 (104.73, 684.49)	-0.039	0.029	-0.001	-0.028	0.187**	0.184**	0.251***	0.053	-0.061	1.000

^1^Values are Pearson correlation coefficients of log_2_-transformed analytes

Abbreviations: AGP, α1-acid glycoprotein

CRP, C-reactive protein

FGF21, fibroblast growth factor 21

FliC, flagellin; I-FABP, intestinal fatty acid-binding protein

Ig, immunoglobulin

IGF-1, insulin-like growth factor 1

IQR, interquartile range

LPS, lipopolysaccharide

OD, optical density

sCD14, soluble CD14

*P < 0.0

**P < 0.01

***P < 0.001

MEEDAT’s EED biomarkers, sCD14 and I-FABP, were moderately correlated with each other (0.367, p<0.001 at 6 weeks and 0.436, p<0.001 at 6 months); sCD14 was also moderately correlated with the systemic inflammation markers, CRP (0.367, p<0.001 at 6 weeks and 0.338, p<0.001 at 6 months) and AGP (0.616, p<0.001 at 6 weeks and 0.410 at 6 months). CRP and AGP were highly correlated with each other (0.715, p<0.001 at 6 weeks and 0.688, p<0.001 at 6 months). Overall, there was a low correlation between anti-flagellin and anti-LPS Igs and MEEDAT biomarkers.

### Associations between biomarkers and growth

**Table 3** shows the associations between quartiles of biomarker concentrations at 6 weeks and subsequent growth outcomes at 12 months. Analyses of quartiles of biomarker concentrations at 6-months are presented in **S2 Table**. In adjusted analyses, higher quartiles of 6-month anti-LPS IgG concentrations were significantly associated with lower LAZ at 12 months (p_trend_ = 0.040) (**S2 Table**). No other significant associations were observed between quartiles of biomarkers concentrations at 6 weeks or 6 months and LAZ, WLZ, or WAZ at 12 months.

**Table 3:** Biomarker concentrations (quartiles) at 6 weeks of age and associations with growth outcomes at 12 months of age^1^.

	Length-for-age *z*-score (LAZ)		Weight-for-length *z*-score (WLZ)		Weight-for-age *z*-score (WAZ)	
	β (95% CI)	*p-trend*	β (95% CI)	*p-trend*	β (95% CI)	*p-trend*
FliC IgA Quartiles (OD)						
1. (< 0.38)	ref	0.147	ref	0.140	ref	0.785
2. (< 0.47)	0.00 (-0.40, 0.41)		0.61 (0.17, 1.04)		0.38 (0.01, 0.75)	
3. (< 0.57)	-0.22 (-0.60, 0.17)		0.46 (-0.02, 0.95)		0.21 (-0.16, 0.58)	
4. (> 0.57)	-0.29 (-0.75, 0.16)		0.34 (-0.12, 0.79)		-0.06 (-0.48, 0.36)	
FliC IgG Quartiles (OD)						
1. (< 0.80)	ref	0.351	ref	0.732	ref	0.153
2. (< 1.00)	-0.01 (-0.42, 0.41)		-0.06 (-0.48, 0.37)		-0.03 (-0.40, 0.34)	
3. (< 1.25)	0.06 (-0.36, 0.48)		-0.17 (-0.64, 0.30)		-0.25 (0.66, 0.17)	
4. (> 1.25)	-0.24 (-0.65, 0.17)		-0.05 (-0.54, 0.45)		-0.25 (-0.67, 0.16)	
LPS IgA Quartiles (OD)						
1. (< 0.23)	ref	0.235	ref	0.274	ref	0.726
2. (< 0.31)	-0.10 (-0.49, 0.28)		0.18 (-0.25, 0.62)		0.06 (-0.29, 0.41)	
3. (< 0.40)	0.00 (-0.40, 0.41)		0.28 (-0.19, 0.75)		0.15 (-0.22, 0.52)	
4. (> 0.40)	-0.31 (-0.71, 0.09)		0.22 (-0.25, 0.68)		-0.14 (-0.55, 0.27)	
LPS IgG Quartiles (OD)						
1. (< 0.61)	ref	0.699	ref	0.536	ref	0.415
2. (< 0.84)	0.05 (-0.35, 0.46)		0.01 (-0.44, 0.45)		0.01 (-0.37, 0.39)	
3. (< 1.11)	-0.02 (-0.41, 0.36)		-0.40 (-0.83, 0.03)		-0.34 (-0.71, 0.03)	
4. (> 1.11)	0.11 (-0.31, 0.54)		0.00 (-0.47, 0.47)		-0.06 (-0.47, 0.36)	
sCD14 Quartiles (ng/mL)						
1. (< 1404.62)	ref	0.084	ref	0.430	ref	0.531
2. (< 1825.50)	-0.28 (-0.70, 0.14)		0.18 (-0.31, 0.67)		-0.04 (-0.42, 0.34)	
3. (< 2287.57)	-0.19 (-0.57, 0.19)		0.15 (0.29, 0.58)		-0.02 (-0.38, 0.34)	
4. (> 2287.57)	-0.44 (-0.89, 0.00)		0.14 (-0.32, 0.59)		-0.25 (-0.67, 0.17)	
I-FABP Quartiles (pg/mL)						
1. (< 732.21)	ref	0.233	ref	0.548	ref	0.568
2. (< 1088.93)	-0.15 (-0.56, 0.26)		0.12 (-0.34, 0.59)		0.15 (-0.24, 0.54)	
3. (< 1634.80)	-0.18 (-0.64, 0.27)		0.27 (-0.21, 0.74)		0.21 (-0.17, 0.59)	
4. (> 1634.80)	-0.34 (-0.76, 0.09)		-0.03 (-0.45, 0.39)		-0.08 (-0.47, 0.32)	
AGP Quartiles (g/L)						
1. (< 0.61)	ref	0.123	ref	0.315	ref	0.666
2. (< 0.83)	0.11 (-0.29, 0.52)		0.22 (-0.20, 0.65)		0.23 (-0.12, 0.58)	
3. (< 1.26)	-0.17 (-0.60, 0.27)		0.23 (-0.27, 0.73)		0.08 (-0.31, 0.47)	
4. (> 1.26)	-0.32 (-0.77, 0.13)		0.23 (-0.23, 0.69)		-0.07 (-0.48, 0.33)	
CRP Quartiles (mg/L)						
1. (< 0.0044)	ref	0.421	ref	0.065	ref	0.676
2. (< 0.36)	0.11 (-0.28, 0.51)		0.10 (-0.33, 0.54)		0.09 (-0.28, 0.46)	
3. (< 4.40)	-0.12 (-0.55, 0.31)		0.24 (-0.22, 0.71)		0.02 (-0.35, 0.40)	
4. (> 4.40)	-0.15 (-0.60, 0.29)		0.42 (-0.03, 0.86)		0.10 (-0.30, 0.51)	
IGF-1 Quartiles (ng/mL)						
1. (< 32.24)	ref	0.181	ref	0.053	ref	0.750
2. (< 66.24)	0.11 (-0.32, 0.55)		-0.35 (-0.78, 0.08)		-0.02 (-0.43, 0.39)	
3. (< 117.92)	0.27 (-0.15, 0.70)		-0.39 (-0.84, 0.06)		0.09 (-0.32, 0.50)	
4. (> 117.92)	0.40 (-0.01, 0.81)		-0.37 (-0.84, 0.11)		0.15 (-0.26, 0.57)	
FGF21 Quartiles (pg/mL)						
1. (< 47.94)	ref	0.340	ref	0.698	ref	0.498
2. (< 88.85)	-0.09 (-0.52, 0.34)		0.14 (-0.29, 0.59)		0.23 (-0.16, 0.61)	
3. (< 174.50)	-0.15 (-0.57, 0.26)		-0.05 (-0.50, 0.41)		0.05 (-0.36, 0.47)	
4. (> 174.50)	-0.30 (-0.70, 0.11)		0.13 (-0.31, 0.56)		0.06 (-0.34, 0.46)	

^1^Models are adjusted for household wealth, maternal age, maternal height, maternal education, maternal marital status, infant sex, infant birth weight, infant age at specimen collection, clinic site, and regimen.

Abbreviations: AGP, α1-acid glycoprotein

CI, confidence interval

CRP, C-reactive protein

FGF21, fibroblast growth factor 21

FliC, flagellin

I-FABP, intestinal fatty acid-binding protein

Ig, immunoglobulin

IGF-1, insulin-like growth factor 1

LPS, lipopolysaccharide

sCD14, soluble CD14

Secondary analyses using log_2_-transformed continuous biomarker concentrations at 6 weeks and 6 months can be found in **S3 Table**. In adjusted analyses, 6-week anti-flagellin IgA concentrations (β: -0.43 z-score, 95% CI: -0.79, -0.07), 6-month anti-LPS IgA concentrations (β: -0.27 z-score, 95% CI: -0.52, -0.01), and 6-month IGF-1 concentrations (β: 0.14 z-score, 95% CI: 0.02, 0.26) were significantly associated with LAZ at 12 months. Furthermore, anti-flagellin IgA (β: -0.35 z-score, 95% CI: -0.63, -0.08), anti-LPS IgA (β: -0.25 z-score, 95% CI: -0.46, -0.05), IGF-1 (β: 0.15 z-score, 95% CI: 0.06, 0.24), and FGF21 (β: -0.07 z-score, 95% CI: -0.13, -0.01) at 6 months were all significantly associated with WAZ at 12 months (**S3 Table**).

### Associations between biomarkers and development

**Table 4** shows the associations between quartiles of biomarker concentrations at 6 weeks and 6 months and subsequent development at 12 months. In adjusted analyses, only higher quartiles of 6-week AGP concentrations were significantly associated with lower CREDI z-scores at 12 months (p_trend_ = 0.026). **S4 Table** shows associations between log_2_-transformed biomarker concentrations at 6 weeks and 6 months and CREDI scores at 12 months, both overall and individually for the domains of motor development, language development, and cognitive development. In adjusted analyses, higher sCD14 concentrations at 6 weeks were significantly associated with lower overall CREDI z-scores (β: -0.29 z-score, 95% CI: -0.57, -0.01), and higher I-FABP concentrations at 6 months were significantly associated with higher overall CREDI z-scores (β: 0.13 z-score 95% CI: 0.03, 0.22).

**Table 4:** Associations between biomarker concentrations (quartiles) at 6 weeks and 6 months of age and overall CREDI z-score at 12 months of age^1^.

	Six-week samples		Six-month samples	
	β (95% CI)	*p-trend*	β (95% CI)	*p-trend*
**FliC IgA Quartiles**				
**1.**	ref	0.353	ref	0.312
**2.**	0.07 (-0.30, 0.45)		0.32 (-0.05, 0.69)	
**3.**	-0.07 (-0.46, 0.33)		0.04 (-0.32, 0.41)	
**4.**	-0.19 (0.64, 0.26)		0.28 (-0.12, 0.68)	
**FliC IgG Quartiles**				
**1.**	ref	0.361	ref	0.205
**2.**	0.20 (-0.14, 0.54)		-0.19 (-0.59, 0.20)	
**3.**	0.21 (-0.22, 0.64)		-0.40 (-0.80, 0.00)	
**4.**	0.16 (-0.19, 0.51)		-0.20 (-0.59, 0.18)	
**LPS IgA Quartiles**				
**1.**	ref	0.118	ref	0.359
**2.**	-0.14 (-0.52, 0.25)		0.03 (-0.35, 0.40)	
**3.**	-0.31 (-0.66, 0.03)		0.15 (-0.21, 0.50)	
**4.**	-0.26 (-0.65, 0.13)		0.15 (-0.24, 0.54)	
**LPS IgG Quartiles**				
**1.**	ref	0.255	ref	0.272
**2.**	0.25 (-0.09, 0.59)		-0.19 (-0.61, 0.23)	
**3.**	0.56 (0.22, 0.90)		-0.33 (-0.75, 0.08)	
**4.**	0.10 (-0.29, 0.50)		-0.18 (-0.57, 0.20)	
**sCD14 Quartiles**				
**1.**	ref	0.485	ref	0.246
**2.**	-0.09 (-0.41, 0.24)		0.23 (-0.12, 0.59)	
**3.**	0.18 (-0.19, 0.55)		0.15 (-0.24, 0.54)	
**4.**	-0.35 (-0.76, 0.07)		0.12 (-0.26, 0.50)	
**1-FABP Quartiles**				
**1.**	ref	0.466	ref	0.381
**2.**	-0.06 (-0.39, 0.27)		0.06 (-0.34, 0.46)	
**3.**	-0.13 (-0.52, 0.27)		0.09 (-0.27, 0.44)	
**4.**	-0.17 (-0.57, 0.23)		0.31 (-0.10, 0.72)	
**AGP Quartiles**				
**1.**	ref	0.026	ref	0.907
**2.**	0.30 (-0.04, 0.64)		0.05 (-0.37, 0.47)	
**3.**	-0.46 (-0.81, -0.11)		-0.05 (-0.43, 0.35)	
**4.**	-0.32 (-0.76, 0.13)		0.06 (-0.35, 0.47)	
**CRP Quartiles**				
**1.**	ref	0.147	ref	0.978
**2.**	-0.02 (-0.37, 0.32)		-0.04 (-0.45, 0.36)	
**3.**	-0.29 (-0.66, 0.09)		-0.23 (-0.61, 0.14)	
**4.**	-0.26 (-0.70, 0.18)		0.09 (-0.26, 0.45)	
**IGF-1 Quartiles**				
**1.**	ref	0.380	ref	0.077
**2.**	0.07 (-0.36, 0.49)		0.30 (-0.05, 0.66)	
**3.**	0.23 (-0.21, 0.67)		0.25 (-0.09, 0.59)	
**4.**	0.25 (-0.15, 0.66)		0.18 (-0.25, 0.62)	
**FGF21 Quartiles**				
**1.**	ref	0.965	ref	0.888
**2.**	0.01 (-0.44, 0.46)		0.15 (-0.22, 0.51)	
**3.**	0.06 (-0.35, 0.46)		-0.09 (-0.47, 0.29)	
**4.**	-0.06 (-0.41, 0.30)		0.00 (-0.39, 0.40)	

^1^Models are adjusted for household wealth, maternal age, maternal height, maternal education, maternal marital status, infant sex, infant birth weight, infant age at specimen collection, clinic site, and regimen.

Abbreviations: AGP, α1-acid glycoprotein; CI, confidence interval; CREDI, Caregiver Reported Early Development Instruments (CREDI); CRP, C-reactive protein; FGF21, fibroblast growth factor 21; FliC, flagellin; I-FABP, intestinal fatty acid-binding protein; Ig, immunoglobulin; IGF-1, insulin-like growth factor 1; LPS, lipopolysaccharide; sCD14, soluble CD14

## Discussion

In this exploratory, prospective cohort study of HEU infants in Dar es Salaam, Tanzania, we examined the relationships between serum biomarkers of EED collected at 6 weeks and 6 months and subsequent growth and development outcomes at 12 months. To our knowledge this is the first study to examine EED specifically in a population of HEU infants, as the vast majority of studies either do not specify the HIV status or use HIV as an exclusion criterion [12]. In primary analyses, higher quartiles of 6-month anti-LPS IgG concentrations were significantly associated with lower LAZ at 12 months, and, in secondary analyses, higher log_2_-transformed 6-week anti-flagellin IgA and 6-month anti-LPS IgA concentrations were significantly associated with lower LAZ at 12 months. No associations were observed between MEEDAT’s EED biomarkers (i.e, I-FABP and sCD14) and infant growth in either primary or secondary analyses. Significant associations were also observed between 6-week AGP, 6-week sCD14, and 6-month I-FABP and CREDI scores at 12 months.

Significant correlations among MEEDAT’s biomarkers were observed in this study, supporting the hypothesis that there are common etiologic pathways between EED, systemic inflammation, and growth hormone resistance [11, 12, 25]. While I-FABP and sCD14 were both positively correlated with AGP, the correlation between sCD14 and AGP was notably stronger at both 6 weeks and 6 months, suggesting microbial translocation, in particular, is a key driver of systemic inflammation. Furthermore, consistent with previous studies, we found CRP and AGP were negatively correlated with IGF-1 [26], although we also found I-FABP and sCD14 to be positively correlated with IGF-1.

Among MEEDAT’s biomarkers, only 6-month, log_2_-tranformed IGF-1 concentrations were found to be significantly associated with lower LAZ at 12 months. These results are consistent with a previous MEEDAT validation study conducted by Arndt et al. which showed no significant association between log_2_-transformed MEEDAT biomarkers, including I-FABP and sCD14, and HAZ over 12 weeks following enrollment in a sample of 300 Malian infants participating in a clinical trial [23]. The study in Mali did, however, find an association between sCD14 and 12-week change in WAZ and WHZ in adjusted analyses. Overall, the current and previous MEEDAT studies are consistent with recent data that have shown no association of I-FABP and sCD14, assessed using conventional ELISAs, with growth faltering, including two studies conducted among Zimbabwean infants [26, 27]. In a study from Northeast Brazil, I-FABP, but not sCD14, was shown to correlate with stunting [28].

Anti-flagellin and anti-LPS Igs indicate a host’s response to microbial translocation, or the passage of bacteria or its components from the lumen of the intestine, across the epithelial barrier, and into the systemic circulation. The results of this study expand upon several previous studies which showed an association between anti-flagellin and anti-LPS Igs and linear growth deficits in infants and young children. Our previous cross-sectional study of 548 HIV-unexposed, uninfected (HUU), 6-months-old, rural Ugandan infants found significant associations between higher anti-flagellin IgA, anti-LPS IgA, and anti-LPS IgG concentrations and lower LAZ [14]. Furthermore, a prospective study of 380 Pakistani children found higher anti-flagellin IgA and anti-LPS IgA at 6 and 9 months were related to declines in linear growth throughout the subsequent 18 months of life [29].

While few studies have examined the association between EED biomarkers and development outcomes in infants and young children, it has been hypothesized that EED and the resulting low-grade systemic inflammation may impact the gut-liver-brain axis and ultimately impair cognitive development [30]. We found that higher sCD14 concentrations at 6 weeks were associated with significantly lower overall development; however, higher I-FABP concentrations at 6 months were associated with higher CREDI scores. This latter finding, that higher antibodies to bacterial components may be positively associated with measures of development, is consistent with a previous cohort of 107 children also from peri-urban Dar es Salaam, Tanzania [15]. That study found a positive association between 6-month anti-LPS IgG, anti-flagellin IgA and IgG concentrations and cognitive scores, 6-month flagellin IgG concentrations and receptive language scores, and 6-month citrulline concentrations and receptive language and gross motor scores at 15 months, all assessed via the Bayley Scales of Infant and Toddler Development- third edition (BSID-III). Though the relationship between EED and child development needs further study, our findings lend credibility to a potential link between pathways involving immune response and neuroprotection.

Our study has strengths and limitations. Specimens for this study were collected as part of a randomized, double-blind, placebo-controlled trial with clear inclusion and exclusion criteria that included HEU infants at high risk for both EED and poor growth and development. Using both conventional ELISA and MEEDAT laboratory methods, we were able to assess multiple domains of EED, systemic inflammation, and growth hormone resistance at two key time points in infancy, 6 weeks and 6 months. Associations between biomarkers and growth and development outcomes were examined using both quartile and log_2_-transformed analyses, controlling for several potential confounders. With regard to limitations, the study included only HEU infants from urban Dar es Salaam, limiting the generalizability of our results. Given the observational nature of the study design, it is important to note that causality cannot be inferred as there may be unmeasured (e.g., diarrheal diseases and intestinal infections) or residual confounding. Furthermore, given the exploratory nature of the study, we did not correct p-values for multiple testing and therefore cannot rule out the possibility that our findings of associations being the result of Type 1 errors (i.e., incorrectly rejecting the null hypothesis). There were also a number of missing serum samples, reducing the study’s sample size and statistical power. Finally, we used a caregiver reported metric to assess child development which may result in some degree of misclassification of child development status.

Overall, we found that unlike anti-flagellin and anti-LPS Igs, MEEDAT’s biomarkers of EED were not associated with linear growth outcomes among HEU infants in Tanzania. In both quartile and continuous analyses, only 6-month IGF-1 showed a significant association with LAZ at 12 months. Thus, although MEEDAT may offer a quick, feasible, and cost-effective way to assess multiple key health and nutrition indicators, its ability to predict children at risk for poor growth and developmental outcomes remains inconclusive. Therefore, with regard to public health practice, we recommended further studies to determine whether the biomarkers examined should be incorporated in the design and evaluation of interventions that address EED in both HEU and HUU populations. Underscoring this is a need for further studies aimed at elucidating the relationships among EED, systemic inflammation, and growth hormone resistance.

Abbreviations: AGP, α1-acid glycoprotein; CREDI, Caregiver Reported Early Development Instruments; CRP, C-reactive protein; EED, environmental enteric dysfunction; ELISA, enzyme-linked immunosorbent assay; FGF21, fibroblast growth factor 21; FliC, flagellin; HEU, HIV-exposed uninfected; HIV, human immunodeficiency virus; I-FABP, intestinal fatty acid-binding protein; Ig, immunoglobulin; IGF-1, insulin-like growth factor 1; LAZ, length-for-age *z*-score; LBW, low birth weight; LMICs, low- and middle-income countries; LPS, lipopolysaccharide; MEEDAT, Micronutrient and EED Assessment Tool; OD, optical density; sCD14, soluble CD14; WAZ, weight-for-age *z*-score; WLZ, weight-for-length *z*-score; WHO, World Health Organization.

## Supporting information

S1 Table Descriptive characteristics of 6-week study sample (n = 365), 6-month study sample (n = 266), and the full EED sub-study sample (n = 720). (DOCX)

S2 Table Biomarker concentrations (quartiles) at 6 months of age and associations with growth outcomes at 12 months of age. (DOCX)

S3 Table Associations between biomarkers concentrations (log_2_-transformed) at 6 weeks and 6 months of age and growth outcomes at 12 months of age. (DOCX)

S4 Table Associations between biomarker concentrations (log_2_-transformed) at 6 weeks and 6 months of age and CREDI z-scores at 12 months of age. (DOCX)

S1 Data Clinical and laboratory data used in manuscript analyses. (DTA)

S1 Strobe Checklist Checklist of items that should be included in reports of cohort studies. (DOCX)
