# Supplementary material for: Assessing environmental enteric dysfunction via multiplex assay and its relation to growth and development among HIV-exposed uninfected Tanzanian infants
Source: PLoS Negl Trop Dis. 2023 Mar 21;17(3):e0011181. doi: 10.1371/journal.pntd.0011181 (PMC10030025; doi:10.1371/journal.pntd.0011181)
Supplement: S1 Table — (DOCX) [file pntd.0011181.s001.docx]

**S1** Descriptive characteristics of 6-week study sample (n=365), 6-month study sample (n=266), and the full EED sub-study sample (n=720)

|  | **6 Week (Mean ± SD or n (%))** | **6 Month (Mean ± SD or n (%))** | **Random (Mean ± SD or n (%))** | |
| --- | --- | --- | --- | --- |
| **Maternal characteristics** |  |  |  |  |
| Age, years |  |  |  |  |
| 18-24 | 54 (14.8) | 35 (13.2) | 107 (14.9) |  |
| 25-35 | 226 (61.9)  8 | 170 (63.9) | 422 (58.6) |  |
| 35+ | 85 (23.3) | 61 (22.9) | 191 (26.5) |  |
| Height, cm | 158.0 ± 6.3 | 158.1 ± 6.5 | 157.8 ± 6.1 |  |
| Education |  |  |  |  |
| No formal education | 45 (12.3) | 34 (12.8)  1 | 82 (11.4) |  |
| Primary | 201 (55.1) | 152 (57.1) | 401 (55.7) |  |
| Secondary/advanced | 118 (32.3) | 79 (29.7) | 236 (32.8) |  |
| Missing | 1 (0.3) | 1 (0.4) | 1 (0.14) |  |
| Marital status |  |  |  |  |
| Married/cohabitating | 281 (77.0) | 205 (77.1) | 553 (76.8) |  |
| Single | 74 (20.3) | 53 (19.9) | 146 (20.3) |  |
| Widowed/divorced/separated | 10 (2.4) | 8 (3.0) | 21 (2.9) |  |
| Regimen |  |  |  |  |
| Placebo | 194 (53.2) | 136 (51.1) | 374 (51.9) |  |
| Vitamin D_3_ | 171 (46.9) | 130 (48.9) | 346 (48.1) |  |
| **Infant characteristics** |  |  |  |  |
| Male | 194 (53.2) | 145 (54.5) | 377 (52.5) |  |
| Low birth weight (<2500 g) | 21 (5.8) | 12 (4.5) | 48/686 (7.0) |  |
| Stunted at 12 months (LAZ < -2) | 143/326 (43.9) | 109/250 (43.6) | 258/565 (45.7) |  |
| Wasted at 12 months (WLZ < -2) | 10/283 (3.5) | 5/238 (2.1) | 13/486 (2.7) |  |
| Underweight at 12 months (WAZ < -2) | 26/286 (9.1)^5^ | 25/242 (10.3) | 42/491 (8.6) |  |

Abbreviations: LAZ, length-for-age *z*-score, WAZ, weight-for-age *z*-score, WLZ, weight-for-length *z*-score
