# Supplementary material for: Assessing environmental enteric dysfunction via multiplex assay and its relation to growth and development among HIV-exposed uninfected Tanzanian infants
Source: PLoS Negl Trop Dis. 2023 Mar 21;17(3):e0011181. doi: 10.1371/journal.pntd.0011181 (PMC10030025; doi:10.1371/journal.pntd.0011181)
Supplement: S2 Table — (DOCX) [file pntd.0011181.s002.docx]

**S2** Biomarker concentrations (quartiles) at 6 months of age and associations with growth outcomes at 12 months of age^1^

|  | Length-for-age *z*-score (LAZ) | | Weight-for-length *z*-score (WLZ) | | Weight-for-age *z*-score (WAZ) | |
| --- | --- | --- | --- | --- | --- | --- |
|  | **β (95% CI)** | ***p-trend*** | **β (95% CI)** | ***p-trend*** | **β (95% CI)** | ***p-trend*** |
| FliC IgA Quartiles (OD) | | | | | | |
| 1. (< 0.54) | ref | 0.269 | ref | 0.080 | ref | 0.063 |
| 2. (< 0.70) | -0.12 (-0.65, 0.41) |  | -0.36 (-0.87, 0.16) |  | -0.24 (-0.65, 0.16) |  |
| 3. (< 0.90) | -0.14 (-0.66, 0.38) |  | -0.37 (-0.87, 0.12) |  | -0.25 (-0.68, 0.19) |  |
| 4. (> 0.90) | -0.30 (-0.82, 0.22) |  | -0.43 (-0.91, 0.05) |  | -0.41 (-0.82, 0.01) |  |
| FliC IgG Quartiles (OD) | | | | | | |
| 1. (< 0.96) | ref | 0.500 | ref | 0.683 | ref | 0.640 |
| 2. (< 1.30) | -0.18 (-0.66, 0.30) |  | -0.07 (-0.60, 0.47) |  | -0.10 (-0.52, 0.31) |  |
| 3. (< 1.80) | -0.01 (-0.50, 0.47) |  | -0.15 (-0.63, 0.33) |  | -0.07 (-0.49, 0.35) |  |
| 4. (> 1.80) | -0.24 (-0.74, 0.26) |  | -0.08 (-0.60, 0.41) |  | -0.12 (-0.54, 0.30) |  |
| LPS IgA Quartiles (OD) | | | | | | |
| 1. (< 0.34) | ref | 0.183 | ref | 0.544 | ref | 0.212 |
| 2. (< 0.45) | 0.18 (-0.35, 0.71) |  | -0.56 (-1.11, -0.01) |  | -0.33 (-0.76, 0.09) |  |
| 3. (< 0.65) | 0.02 (-0.45, 0.49) |  | -0.11 (-0.59, 0.37) |  | -0.03 (-0.43, 0.37) |  |
| 4. (> 0.65) | -0.34 (-0.84, 0.15) |  | -0.26 (-0.74, 0.22) |  | -0.35 (-0.75, 0.05) |  |
| LPS IgG Quartiles (OD) | | | | | | |
| 1. (< 0.78) | ref | **0.040** | ref | 0.887 | ref | 0.340 |
| 2. (< 1.18) | -0.59 (-1.10, -0.07) |  | -0.15 (-0.67, 0.36) |  | -0.36 (-0.76, 0.04) |  |
| 3. (< 1.71) | -0.33 (-0.85, 0.19) |  | -0.03 (-0.52, 0.45) |  | -0.17 (-0.57, 0.24) |  |
| 4. (> 1.71) | -0.66 (-1.19, -0.12) |  | -0.07 (-0.61, 0.46) |  | -0.27 (-0.69, 0.15) |  |
| sCD14 Quartiles (ng/mL) | | | | | | |
| 1. (< 1683.76) | ref | 0.424 | ref | 0.355 | ref | 0.963 |
| 2. (< 2200.07) | 0.03 (-0.44, 0.50) |  | -0.11 (-0.62, 0.40) |  | 0.02 (-0.38, 0.41) |  |
| 3. (< 2876.08) | 0.22 (-0.31, 0.76) |  | -0.09 (-0.53, 0.36) |  | 0.07 (-0.34, 0.48) |  |
| 4. (> 2876.08) | 0.25 (-0.26, 0.75) |  | -0.36 (-0.83, 0.11) |  | -0.13 (-0.55, 0.29) |  |
| I-FABP Quartiles (pg/mL) | | | | | | |
| 1. (< 411.72) | ref | 0.101 | ref | 0.955 | ref | 0.333 |
| 2. (< 564.95) | -0.33 (-0.86, 0.21) |  | -0.18 (-0.69, 0.34) |  | -0.28 (-0.69, 0.13) |  |
| 3. (< 856.65) | -0.33 (-0.83, 0.18) |  | 0.12 (-0.44, 0.69) |  | -0.08 (-0.54, 0.37) |  |
| 4. (> 856.65) | -0.47 (-1.01, 0.06) |  | 0.11 (-0.38, 0.59) |  | -0.17 (-0.59, 0.24) |  |
| AGP Quartiles (g/L) | | | | | | |
| 1. (< 0.83) | ref | 0.867 | ref | 0.390 | ref | 0.753 |
| 2. (< 1.05) | 0.10 (-0.38, 0.58) |  | 0.47 (-0.04, 0.99) |  | 0.41 (0.02, 0.81) |  |
| 3. (< 1.43) | 0.16 (-0.36, 0.69) |  | -0.10 (-0.62, 0.41) |  | -0.03 (-0.41, 0.35) |  |
| 4. (> 1.43) | -0.07 (-0.60, 0.45) |  | 0.40 (-0.09, 0.90) |  | 0.20 (-0.22, 0.61) |  |
| CRP Quartiles (mg/L) | | | | | | |
| 1. (< 0.08) | ref | 0.179 | ref | 0.909 | ref | 0.568 |
| 2. (< 0.29) | 0.11 (-0.39, 0.62) |  | 0.04 (-0.47, 0.55) |  | 0.10 (-0.28, 0.47) |  |
| 3. (< 1.54) | 0.29 (-0.16, 0.73) |  | 0.16 (-0.30, 0.62) |  | 0.28 (-0.12, 0.68) |  |
| 4. (> 1.54) | 0.29 (-0.20, 0.79) |  | -0.10 (-0.55, 0.35) |  | 0.03 (-0.37, 0.42) |  |
| IGF-1 Quartiles (ng/mL) | | | | | | |
| 1. (< 9.64) | ref | 0.432 | ref | 0.637 | ref | 0.294 |
| 2. (< 20.63) | 0.27 (-0.23, 0.78) |  | 0.05 (-0.48, 0.58) |  | 0.15 (-0.23, 0.53) |  |
| 3. (< 44.52) | 0.14 (-0.32, 0.60) |  | 0.26 (-0.21, 0.74) |  | 0.35 (-0.06, 0.75) |  |
| 4. (> 44.52) | 0.62 (0.09, 1.15) |  | 0.33 (-0.16, 0.81) |  | 0.57 (0.17, 0.98) |  |
| FGF21 Quartiles (pg/mL) | | | | | | |
| 1. (< 104.73) | ref | 0.693 | ref | 0.654 | ref | 0.544 |
| 2. (< 269.61) | -0.02 (-0.52, 0.49) |  | 0.02 (-0.45, 0.48) |  | 0.02 (-0.40, 0.45) |  |
| 3. (< 684.49) | 0.02 (-0.49, 0.53) |  | 0.02 (-0.46, 0.50) |  | 0.06 (-0.36, 0.48) |  |
| 4. (> 684.49) | -0.32 (-0.83, 0.20) |  | -0.38 (-0.88, 0.13) |  | -0.51 (-0.93, -0.09) |  |

^1^Models are adjusted for household wealth, maternal age, maternal height, maternal education, maternal marital status, infant sex, infant birth weight, infant age at specimen collection, clinic site, and regimen.

Abbreviations: AGP, α1-acid glycoprotein; CI, confidence interval; CRP, C-reactive protein; FGF21, fibroblast growth factor 21; FliC, flagellin; I-FABP, intestinal fatty acid-binding protein; Ig, immunoglobulin; IGF-1, insulin-like growth factor 1; LPS, lipopolysaccharide; sCD14, soluble CD14
