# Supplementary material for: Assessing environmental enteric dysfunction via multiplex assay and its relation to growth and development among HIV-exposed uninfected Tanzanian infants
Source: PLoS Negl Trop Dis. 2023 Mar 21;17(3):e0011181. doi: 10.1371/journal.pntd.0011181 (PMC10030025; doi:10.1371/journal.pntd.0011181)
Supplement: S3 Table — (DOCX) [file pntd.0011181.s003.docx]

**S3** Associations between biomarkers concentrations (log_2_-transformed) at 6 weeks and 6 months of age and growth outcomes at 12 months of age^1^

|  |  | **Six-week samples** | | | | **Six-month samples** | | | |
| --- | --- | --- | --- | --- | --- | --- | --- | --- | --- |
|  |  | **Crude β (95%CI)** | ***p-value*** | **Adjusted β**  **(95% CI)** | ***p-value*** | **Crude β (95%CI)** | **p-value** | **Adjusted β**  **(95% CI)** | ***p-value*** |
| **LAZ** | **FliC IgA** | -0.28 (-0.64, 0.08) | 0.123 | -0.43 (-0.79, -0.07) | **0.021** | -0.31 (-0.72, 0.09) | 0.130 | -0.32 (-0.68, 0.04) | 0.083 |
|  | **FliC IgG** | -0.10 (-0.39, 0.19) | 0.502 | -0.17 (-0.44, 0.10) | 0.211 | -0.09 (-0.38, 0.20) | 0.542 | -0.18 (-0.47, 0.10) | 0.206 |
|  | **LPS IgA** | -0.10 (-0.35, 0.16) | 0.463 | -0.09 (-0.34, 0.15) | 0.455 | -0.29 (-0.59, 0.00) | **0.047** | -0.27 (-0.52, -0.01) | **0.039** |
|  | **LPS IgG** | 0.08 (-0.17, 0.33) | 0.538 | 0.02 (-0.21, 0.25) | 0.871 | -0.17 (-0.41, 0.07) | 0.162 | -0.22 (-0.44, 0.01) | 0.062 |
|  | **sCD14** | -0.08 (-0.28, 0.12) | 0.437 | -0.10 (-0.30, 0.10) | 0.316 | 0.09 (-0.20, 0.38) | 0.524 | 0.10 (-0.18, 0.38) | 0.499 |
|  | **I-FABP** | -0.14 (-0.29, 0.02) | 0.084 | -0.10 (-0.24, 0.05) | 0.198 | -0.12 (-0.33, 0.08) | 0.231 | -0.05 (-0.23, 0.14) | 0.604 |
|  | **AGP** | -0.21 (-0.41, 0.00) | **0.049** | -0.20 (-0.42, 0.02) | 0.073 | -0.16 (-0.43, 0.11) | 0.257 | -0.15 (-0.42, 0.13) | 0.302 |
|  | **CRP** | -0.03 (-0.06, 0.01) | 0.167 | -0.02 (-0.05, 0.02) | 0.397 | 0.00 (-0.05, 0.05) | 0.860 | 0.01 (-0.04, 0.06) | 0.623 |
|  | **IGF-1** | 0.05 (-0.06, 0.16) | 0.391 | 0.06 (-0.05, 0.16) | 0.312 | 0.07 (-0.05, 0.19) | 0.271 | 0.14 (0.02, 0.26) | **0.019** |
|  | **FGF21** | -0.10 (-0.21, 0.00) | **0.047** | -0.07 (-0.16, 0.02) | 0.110 | -0.01 (-0.08, 0.06) | 0.795 | -0.04 (-0.11, 0.04) | 0.343 |
| **WLZ** | **FliC IgA** | 0.19 (-0.19, 0.57) | 0.327 | 0.27 (-0.10, 0.64) | 0.157 | -0.34 (-0.69, 0.01) | 0.054 | -0.32 (-0.66, 0.00) | 0.053 |
|  | **FliC IgG** | -0.06 (-0.39, 0.28) | 0.739 | -0.01 (-0.34, 0.33) | 0.960 | -0.14 (-0.40, 0.12) | 0.305 | -0.06 (-0.33, 0.21) | 0.674 |
|  | **LPS IgA** | 0.14 (-0.11, 0.39) | 0.284 | 0.13 (-0.12, 0.39) | 0.299 | -0.16 (-0.42, 0.10) | 0.218 | -0.17 (-0.41, 0.07) | 0.163 |
|  | **LPS IgG** | 0.00 (-0.26, 0.25) | 0.977 | 0.05 (-0.19, 0.28) | 0.708 | -0.07 (-0.29, 0.15) | 0.524 | 0.01 (-0.21, 0.23) | 0.944 |
|  | **sCD14** | -0.10 (-0.36, 0.16) | 0.471 | -0.05 (-0.31, 0.21) | 0.722 | -0.11 (-0.38, 0.15) | 0.400 | -0.16 (-0.42, 0.10) | 0.239 |
|  | **I-FABP** | 0.00 (-0.18, 0.18) | 0.975 | -0.04 (-0.21, 0.13) | 0.642 | 0.01 (-0.13, 0.15) | 0.882 | -0.04 (-0.18, 0.10) | 0.564 |
|  | **AGP** | 0.06 (-0.16, 0.27) | 0.615 | 0.07 (-0.15, 0.30) | 0.532 | 0.09 (-0.19, 0.36) | 0.533 | 0.11 (-0.15, 0.36) | 0.416 |
|  | **CRP** | 0.04 (0.01, 0.08) | **0.014** | 0.03 (-0.01, 0.07) | 0.095 | 0.01 (-0.04, 0.06) | 0.727 | 0.00 (-0.04, 0.05) | 0.916 |
|  | **IGF-1** | -0.05 (-0.17, 0.07) | 0.388 | -0.07 (-0.19, 0.06) | 0.295 | 0.12 (0.01, 0.22) | **0.026** | 0.09 (-0.01, 0.20) | 0.080 |
|  | **FGF21** | -0.01 (-0.12, 0.09) | 0.795 | -0.01 (-0.11, 0.08) | 0.824 | -0.09 (-0.16, -0.02) | **0.010** | -0.07 (-0.14, 0.00) | 0.066 |
| **WAZ** | **FliC IgA** | -0.15 (-0.51, 0.22) | 0.436 | -0.18 (-0.56, 0.19) | 0.338 | -0.36 (-0.66, -0.06) | **0.020** | -0.35 (-0.63, -0.08) | **0.011** |
|  | **FliC IgG** | -0.14 (-0.40, 0.11) | 0.267 | -0.19 (-0.45, 0.08) | 0.170 | -0.12 (-0.34, 0.100 | 0.280 | -0.10 (-0.34, 0.13) | 0.390 |
|  | **LPS IgA** | -0.04 (-0.30, 0.21) | 0.738 | -0.05 (-0.30, 0.21) | 0.714 | -0.25 (-0.49, -0.02) | **0.034** | -0.25 (-0.46, -0.05) | **0.015** |
|  | **LPS IgG** | -0.02 (-0.25, 0.21) | 0.862 | -0.03 (-0.26, 0.19) | 0.777 | -0.09 (-0.29, 0.10) | 0.341 | -0.06 (-0.25, 0.14) | 0.556 |
|  | **sCD14** | -0.15 (-0.39, 0.10) | 0.237 | -0.17 (-0.42, 0.08) | 0.171 | -0.04 (-0.24, 0.17) | 0.734 | -0.07 (-0.30, 0.16) | 0.540 |
|  | **I-FABP** | -0.07 (-0.23, 0.10) | 0.429 | -0.06 (-0.22, 0.11) | 0.507 | -0.05 (-0.15, 0.04) | 0.280 | -0.06 (-0.16, 0.04) | 0.264 |
|  | **AGP** | -0.08 (-0.26, 0.10) | 0.371 | -0.10 (-0.29, 0.09) | 0.308 | -0.02 (-0.26, 0.21) | 0.839 | -0.03 (-0.26, 0.21) | 0.827 |
|  | **CRP** | 0.01 (-0.02, 0.04) | 0.515 | 0.00 (-0.03, 0.04) | 0.902 | 0.00 (-0.03, 0.04) | 0.818 | 0.00 (-0.04, 0.04) | 0.864 |
|  | **IGF-1** | 0.04 (-0.06, 0.15) | 0.432 | 0.04 (-0.07, 0.14) | 0.476 | 0.13 (0.04, 0.22) | **0.004** | 0.15 (0.06, 0.24) | **0.001** |
|  | **FGF21** | -0.05 (-0.13, 0.03) | 0.260 | -0.03 (-0.11, 0.06) | 0.531 | -0.07 (-0.12, -0.01) | **0.015** | -0.07 (-0.13, -0.01) | **0.016** |

^1^Adjusted models control for household wealth, maternal age, maternal height, maternal education, maternal marital status, infant sex, infant birth weight, infant age at specimen collection, clinic site, and regimen

Abbreviations: AGP, α1-acid glycoprotein; CI, confidence interval; CRP, C-reactive protein; FGF21, fibroblast growth factor 21; FliC, flagellin; I-FABP, intestinal fatty acid-binding protein; Ig, immunoglobulin; IGF-1, insulin-like growth factor 1; LAZ, length-for-age *z*-score; LPS, lipopolysaccharide; OD, optical density; sCD14, soluble CD14; WAZ, weight-for-age *z*-score, WLZ, weight-for-length *z*-score
