# Supplementary material for: Assessing environmental enteric dysfunction via multiplex assay and its relation to growth and development among HIV-exposed uninfected Tanzanian infants
Source: PLoS Negl Trop Dis. 2023 Mar 21;17(3):e0011181. doi: 10.1371/journal.pntd.0011181 (PMC10030025; doi:10.1371/journal.pntd.0011181)
Supplement: S4 Table — (DOCX) [file pntd.0011181.s004.docx]

**S4** Associations between biomarker concentrations (log_2_-transformed) at 6 weeks and 6 months of age and CREDI z-scores at 12 months of age^1^

|  | **Motor** | | **Language** | | **Cognitive** | | **Overall** | |
| --- | --- | --- | --- | --- | --- | --- | --- | --- |
| **Six-week samples** | | | | | | | | |
|  | **Adjusted β (95%CI)** | ***p-value*** | **Adjusted β**  **(95% CI)** | ***p-value*** | **Adjusted β (95%CI)** | ***p-value*** | **Adjusted β**  **(95% CI)** | ***p-value*** |
| **FliC IgA** | -0.33 (-0.69, 0.03) | 0.075 | -0.22 (-0.63, 0.20) | 0.309 | -0.21 (-0.61, 0.20) | 0.315 | -0.30 (-0.70, 0.10) | 0.136 |
| **FliC IgG** | 0.19 (-0.04, 0.42) | 0.097 | -0.01 (-0.29, 0.27) | 0.933 | 0.03 (-0.20, 0.27) | 0.780 | 0.12 (-0.12, 0.35) | 0.337 |
| **LPS IgA** | -0.23 (-0.48, 0.03) | 0.080 | -0.20 (-0.47, 0.08) | 0.150 | -0.15 (-0.42, 0.13) | 0.298 | -0.22 (-0.50, 0.05) | 0.105 |
| **LPS IgG** | 0.15 (-0.07, 0.37) | 0.185 | 0.00 (-0.25, 0.25) | 0.986 | 0.07 (-0.16, 0.31) | 0.526 | 0.08 (-0.15, 0.31) | 0.514 |
| **sCD14** | -0.29 (-0.58, 0.00) | 0.051 | -0.27 (-0.55, 0.00) | 0.053 | -0.29 (-0.61, 0.02) | 0.070 | -0.29 (-0.57, -0.01) | **0.042** |
| **I-FABP** | -0.08 (-0.24, 0.08) | 0.317 | -0.05 (-0.18, 0.08) | 0.432 | 0.00 (-0.14, 0.13) | 0.947 | -0.08 (-0.22, 0.06) | 0.258 |
| **AGP** | -0.19 (-0.39, 0.02) | 0.070 | -0.13 (-0.35, 0.08) | 0.230 | -0.16 (-0.37, 0.06) | 0.154 | -0.17 (-0.37, 0.02) | 0.084 |
| **CRP** | -0.04 (-0.07, 0.00) | **0.043** | -0.02 (-0.06, 0.02) | 0.267 | -0.03 (-0.07, 0.01) | 0.118 | -0.03 (-0.07, 0.00) | 0.091 |
| **IGF-1** | 0.01 (-0.10, 0.11) | 0.896 | 0.07 (-0.05, 0.19) | 0.262 | 0.03 (-0.08, 0.14) | 0.614 | 0.03 (-0.08, 0.14) | 0.607 |
| **FGF21** | -0.01 (-0.09, 0.06) | 0.757 | -0.02 (-0.10, 0.06) | 0.678 | 0.01 (-0.07, 0.08) | 0.846 | -0.01 (-0.08, 0.06) | 0.855 |
| **Six-month samples** | | | | | | | | |
|  |  |  |  |  |  |  |  |  |
| **FliC IgA** | 0.07 (-0.16, 0.29) | 0.563 | 0.11 (-0.23, 0.44) | 0.525 | 0.03 (-0.22, -0.29) | 0.803 | 0.09 (-0.17, 0.34) | 0.493 |
| **FliC IgG** | -0.05 (-0.26, 0.16) | 0.653 | -0.03 (-0.34, 0.27) | 0.829 | -0.03 (-0.26, 0.21) | 0.808 | -0.07 (-0.30, 0.16) | 0.537 |
| **LPS IgA** | 0.04 (-0.14, 0.23) | 0.635 | -0.01 (-0.27, 0.26) | 0.965 | 0.03 (-0.17, 0.24) | 0.747 | 0.02 (-0.18, 0.22) | 0.835 |
| **LPS IgG** | -0.05 (-0.23, 0.12) | 0.552 | -0.09 (-0.35, 0.17) | 0.489 | -0.09 (-0.28, 0.11) | 0.395 | -0.09 (-0.28, 0.10) | 0.372 |
| **sCD14** | -0.02 (-0.25, 0.20) | 0.847 | 0.29 (0.02, 0.57) | **0.037** | 0.18 (-0.05, 0.42) | 0.118 | 0.13 (-0.09, 0.34) | 0.259 |
| **I-FABP** | 0.09 (-0.02, 0.19) | 0.104 | 0.19 (0.07, 0.31) | **0.002** | 0.16 (0.07, 0.26) | **0.001** | 0.13 (0.03, 0.22) | **0.008** |
| **AGP** | -0.12 (-0.30, 0.07) | 0.228 | -0.01 (-0.26, 0.24) | 0.931 | -0.10 (-0.35, 0.14) | 0.415 | -0.10 (-0.31, 0.11) | 0.339 |
| **CRP** | -0.01 (-0.05, 0.02) | 0.400 | 0.00 (-0.04, 0.04) | 0.981 | -0.02 (-0.05, 0.02) | 0.409 | -0.01 (-0.05, 0.03) | 0.560 |
| **IGF-1** | 0.03 (-0.06, 0.12) | 0.534 | 0.02 (-0.11, 0.14) | 0.798 | 0.04 (-0.06, 0.14) | 0.451 | 0.04 (-0.06, 0.14) | 0.406 |
| **FGF21** | 0.02 (-0.04, 0.07) | 0.568 | -0.02 (-0.09, 0.05) | 0.529 | 0.02 (-0.04, 0.07) | 0.498 | 0.01 (-0.05, 0.06) | 0.799 |

^1^Models are adjusted for household wealth, maternal age, maternal height, maternal education, maternal marital status, infant sex, infant birth weight, infant age at specimen collection, clinic site, and regimen.

Abbreviations: AGP, α1-acid glycoprotein; CI, confidence interval; CRP, C-reactive protein; FGF21, fibroblast growth factor 21; FliC, flagellin; I-FABP, intestinal fatty acid-binding protein; Ig, immunoglobulin; IGF-1, insulin-like growth factor 1; LPS, lipopolysaccharide; sCD14, soluble CD14
