# Supplementary material for: Assessing environmental enteric dysfunction via multiplex assay and its relation to growth and development among HIV-exposed uninfected Tanzanian infants
Source: PLoS Negl Trop Dis. 2023 Mar 21;17(3):e0011181. doi: 10.1371/journal.pntd.0011181 (PMC10030025; doi:10.1371/journal.pntd.0011181)
Supplement: S1 Strobe Checklist — (DOCX) [file pntd.0011181.s006.docx]

**S1** STROBE Checklist—Checklist of items that should be included in reports of ***cohort studies***

|  | **Item No** | **Recommendation** |  |
| --- | --- | --- | --- |
| **Title and abstract** | 1 | (*a*) Indicate the study’s design with a commonly used term in the title or the abstract Wording makes clear it is a cohort to a trial |  |
|  |  | (*b*) Provide in the abstract an informative and balanced summary of what was done and what was found Done, see abstract |  |
| **Introduction** | | |  |
| Background/rationale | 2 | Explain the scientific background and rationale for the investigation being reported Done, see introduction re burden of stunting, EED and need for improved markers |  |
| Objectives | 3 | State specific objectives, including any prespecified hypotheses  The primary goal of this study was to examine the relationship between biomarkers of EED, including anti-flagellin and anti-LPS IgA and IgG as well as those in MEEDAT, and growth and developmental outcomes among HEU Tanzanian infants at risk of EED. |  |
| **Methods** | | |  |
| Study design | 4 | Present key elements of study design early in the paper Done, see methods |  |
| Setting | 5 | Describe the setting, locations, and relevant dates, including periods of recruitment, exposure, follow-up, and data collection Done, see methods |  |
| Participants | 6 | (*a*) Give the eligibility criteria, and the sources and methods of selection of participants. Describe methods of follow-up Selection criteria for parent trial and sub-study given |  |
|  |  | (*b*) For matched studies, give matching criteria and number of exposed and unexposed N/A |  |
| Variables | 7 | Clearly define all outcomes, exposures, predictors, potential confounders, and effect modifiers. Give diagnostic criteria, if applicable Done, see pg 9 |  |
| Data sources/ measurement | 8* | For each variable of interest, give sources of data and details of methods of assessment (measurement). Describe comparability of assessment methods if there is more than one group Done, see pg 9 |  |
| Bias | 9 | Describe any efforts to address potential sources of bias See methods and discussion |  |
| Study size | 10 | Explain how the study size was arrived at Sample size calculation included |  |
| Quantitative variables | 11 | Explain how quantitative variables were handled in the analyses. If applicable, describe which groupings were chosen and why See pg 9 |  |
| Statistical methods | 12 | (*a*) Describe all statistical methods, including those used to control for confounding See pg 9-10 |  |
|  |  | (*b*) Describe any methods used to examine subgroups and interactions Primary and secondary analyses conducted at 2 time points |  |
|  |  | (*c*) Explain how missing data were addressed Sample sizes included |  |
|  |  | (*d*) If applicable, explain how loss to follow-up was addressed N/A |  |
|  |  | (*e*) Describe any sensitivity analyses N/A |  |
| **Results** | | |  |
| Participants | 13* | (a) Report numbers of individuals at each stage of study—eg numbers potentially eligible, examined for eligibility, confirmed eligible, included in the study, completing follow-up, and analysed |  |
|  |  | (b) Give reasons for non-participation at each stage  Flow diagram gives reason for non-participation at each stage |  |
|  |  | (c) Consider use of a flow diagram  Included |  |
| Descriptive data | 14* | (a) Give characteristics of study participants (eg demographic, clinical, social) and information on exposures and potential confounders Done, see Table 1 |  |
|  |  | (b) Indicate number of participants with missing data for each variable of interest Done, see Table 1 and Supplementary Figure 1 |  |
|  |  | (c) Summarise follow-up time (eg, average and total amount) Done |  |
| Outcome data | 15* | Report numbers of outcome events or summary measures over time Done |  |
| Main results | 16 | (*a*) Give unadjusted estimates and, if applicable, confounder-adjusted estimates and their precision (eg, 95% confidence interval). Make clear which confounders were adjusted for and why they were included Done |  |
|  |  | (*b*) Report category boundaries when continuous variables were categorized Done |  |
|  |  | (*c*) If relevant, consider translating estimates of relative risk into absolute risk for a meaningful time period N/A |  |
| Other analyses | 17 | Report other analyses done—eg analyses of subgroups and interactions, and sensitivity analyses  Both primary analyses (quartiles) and secondary analyses (continuous) were described |  |
| **Discussion** | | |  |
| Key results | 18 | Summarise key results with reference to study objectives  See discussion section (pg 13) |  |
| Limitations | 19 | Discuss limitations of the study, taking into account sources of potential bias or imprecision. Discuss both direction and magnitude of any potential bias  See pg 15-16 |  |
| Interpretation | 20 | Give a cautious overall interpretation of results considering objectives, limitations, multiplicity of analyses, results from similar studies, and other relevant evidence  Done, see discussion section and concluding paragraph |  |
| Generalisability | 21 | Discuss the generalisability (external validity) of the study results  Done, see discussion section (pg 15) |  |
| **Other information** | | |  |
| Funding | 22 | Give the source of funding and the role of the funders for the present study and, if applicable, for the original study on which the present article is based Funding source of parent trial and sub study included |  |

*Give information separately for exposed and unexposed groups.
